# Surgical correction of total anomalous pulmonary venous connection to persistent left-sided superior vena cava: a case report

**DOI:** 10.1093/ehjcr/ytae290

**Published:** 2024-07-26

**Authors:** Uday Tej, Anand Kumar Mishra, Apeksha Mittal, Kulbhushan Saini, Arun George

**Affiliations:** Department of Cardiothoracic and Vascular Surgery, Post Graduate Institute of Medical Education and Research, Chandigarh 160015, India; Department of Cardiothoracic and Vascular Surgery, Post Graduate Institute of Medical Education and Research, Chandigarh 160015, India; Department of Cardiothoracic and Vascular Surgery, Post Graduate Institute of Medical Education and Research, Chandigarh 160015, India; Department of Anesthesia and Intensive Care, Post Graduate Institute of Medical Education and Research, Chandigarh, India; Department of Cardiothoracic and Vascular Surgery, Post Graduate Institute of Medical Education and Research, Chandigarh 160015, India

**Keywords:** Case report, Total anomalous pulmonary venous connection, Double outlet right ventricle, Persistent left-sided vena cava, Single ventricle physiology

## Abstract

**Background:**

Total anomalous pulmonary venous connection (TAPVC) to left superior vena cava (LSVC) is an extremely rare congenital heart disease, and its surgical management is very challenging.

**Case summary:**

We report one such case of a 5-year-old south Asian male with double outlet right ventricle and unbalanced atrioventricular canal defect, where all the pulmonary veins were found opening into LSVC, which was then opening into the left side of the common atrium. Intraoperatively, the LSVC was transected just below the left internal jugular vein and left subclavian vein junction and left-sided bidirectional Glenn shunt done using 8 mm Dacron tube graft. Pulmonary veins were left draining through the LSVC into the common atrium. Right-sided Glenn shunt was completed as usual. Currently, the patient is year and half post-surgery and is doing well; school going on par with the peer group maintaining a room air saturation of 87%.

**Discussion:**

Here, we report a successful surgical correction of TAPVC to LSVC in a child with univentricular physiology, however due to the paucity of data and rarity of such cases, optimal surgical management is yet to be defined.

**Learning points**

- A rarity that a complex congenital heart disease with the development of all four pulmonary veins draining into persistent left superior vena cava, double outlet right ventricle, pulmonary stenosis, and complete AV canal defect.
- Surgical challenges are faced to mobilize all the structures for performing a bilateral bidirectional Glenn shunt, especially the left-sided Glenn shunt using a Dacron tube graft.

## Introduction

Total anomalous pulmonary venous connection (TAPVC) represents 1–3% of all congenital heart diseases.^1^ Embryologically, the pulmonary venous plexus, surrounding the lung bud, normally establishes connections with pulmonary veins originating from the dorsal wall of the sinus venosus. As development progresses, these connections should disappear concerning the cardinal and umbilicovitelline veins. The abnormal persistence of these connections results in the formation of TAPVC.^2^ In rare instances, when these connections persist abnormally along with the left pre-cardinal vein, an unusual configuration occurs, leading to anomalous (total or partial) pulmonary venous drainage into the persistent left superior vena cava (LSVC).^3^ Sinha *et al*.^4^ reported findings from CT angiography in a case with TAPVC to LSVC, coexisting with double outlet right ventricle (DORV), ventricular septal defect (VSD), and pulmonary stenosis (PS). In this report, we present a case involving a DORV and an unbalanced atrioventricular canal defect (AVCD). A peculiar finding, in this case, was the discovery that all pulmonary veins were draining into the LSVC, which was subsequently opening into the left side of the common atrium that was not reported in the literature. This case highlights the importance of thorough diagnostic evaluations to identify complex cardiac anomalies and underscores the need for individualized management strategies. In this presentation, we detail the surgical correction of a case involving a univentricular heart with this unique anatomy.

## Summary figure

**Figure:**
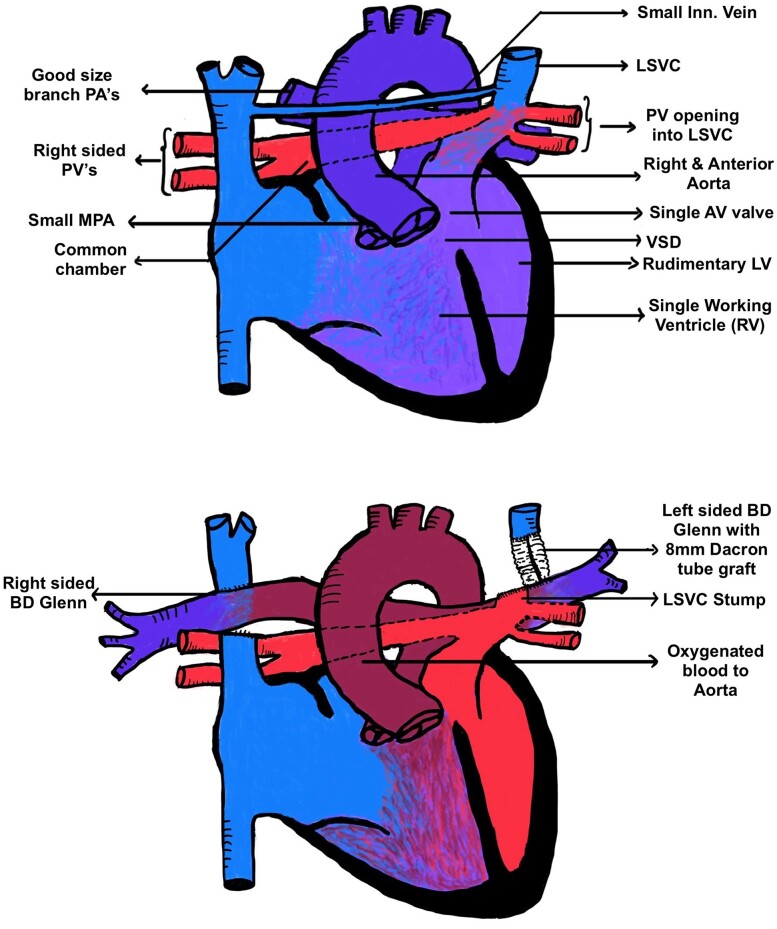


## Case summary

A pre-school 5-year-old south Asian boy born out of non-consanguinity presented to the medical outpatient department with complaints of constant progressive cyanosis associated with dyspnoea on exertion since 3 years of age, progressed to current NYHA grade II at presentation. No history is suggestive of inborn metabolic disorder. Their family belongs to low socio-economic status.

On general examination, he had no gross dysmorphism with preserved growth (W/A at 3rd centile, H/A at 5th centile, and W/H at 7th centile) motor developmental delay with central cyanosis and grade 3 pan digital clubbing with room air saturation of 55–60%, with mild tachypnoea and normal symmetrical chest, with the apex at 5th intercostal space midclavicular line, with no cardiomegaly on percussion. On auscultation, there was a soft S1, prominent A2, moderate intensity ejection systolic murmur detected in the left parasternal border in 2nd and 3rd intercostal space, and normal vesicular breath sounds in bilateral lungs.

**Figure 1:**
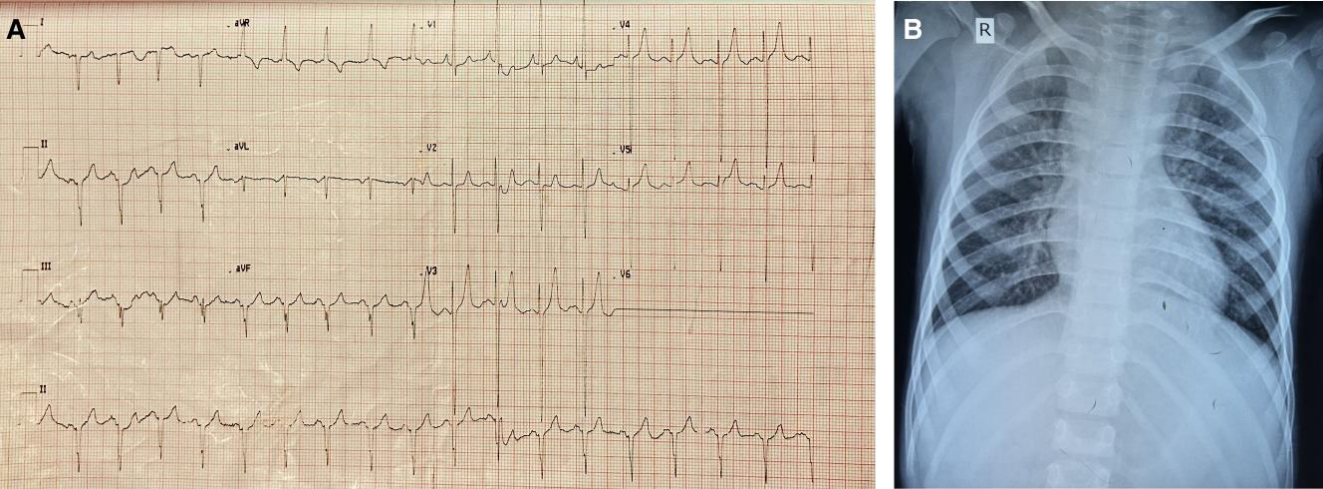
(*A*) Electrocardiogram of the patient. (*B*) Chest X-ray of the patient showing plethoric lung fields.

**Figure 2:**
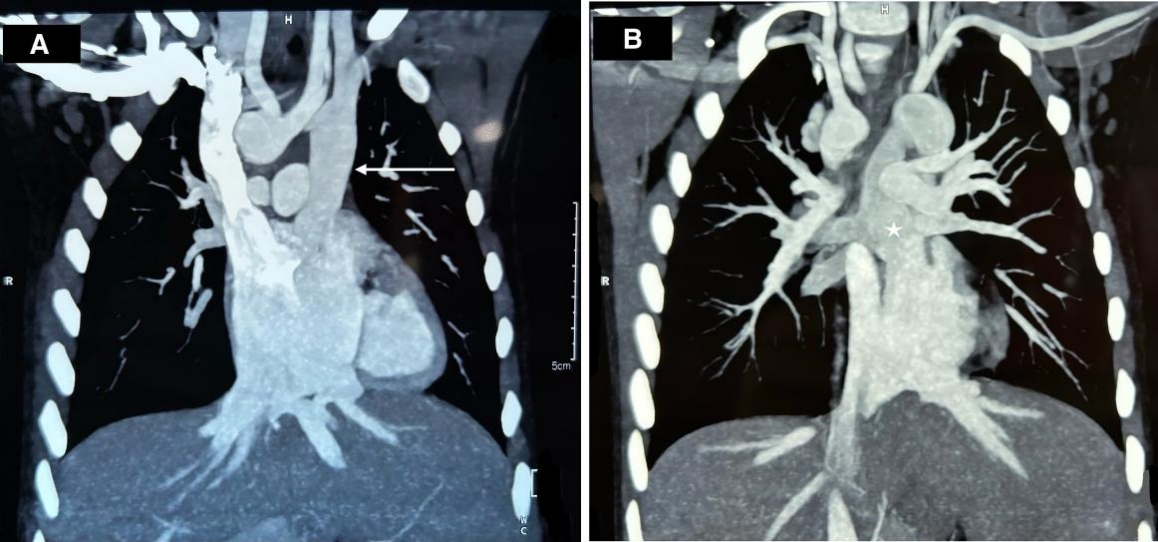
Cardiac CT of the patient showing (*A*) LSVC draining into the left side of the common atrium (arrow) and (*B*) pulmonary veins draining into the LSVC (star).

The haematological assessment revealed haemoglobin—18.9 g/dL, haematocrit—63.3, and platelet count—288 × 109. The electrocardiogram revealed extreme right axis deviation, right ventricular predominance, and diminished left ventricular force (*Figure 1A*). The chest X-ray displayed plethoric lung fields (*Figure 1B*). Transthoracic echocardiogram was suggestive of supra cardiac TAPVC, unbalanced complete AVCD, rudimentary left ventricle, single atrioventricular valve with mild atrioventricular valve regurgitation (AVVR), DORV, VSD, PS, and malposed great vessels. Cardiac computed tomography (CT) revealed all four pulmonary veins coalescing and draining into LSVC and then to the left side of the common atrium, along with DORV, AVCD, and right-sided aortic arch (*Figure 2A*, and *B*).

**Figure 3:**
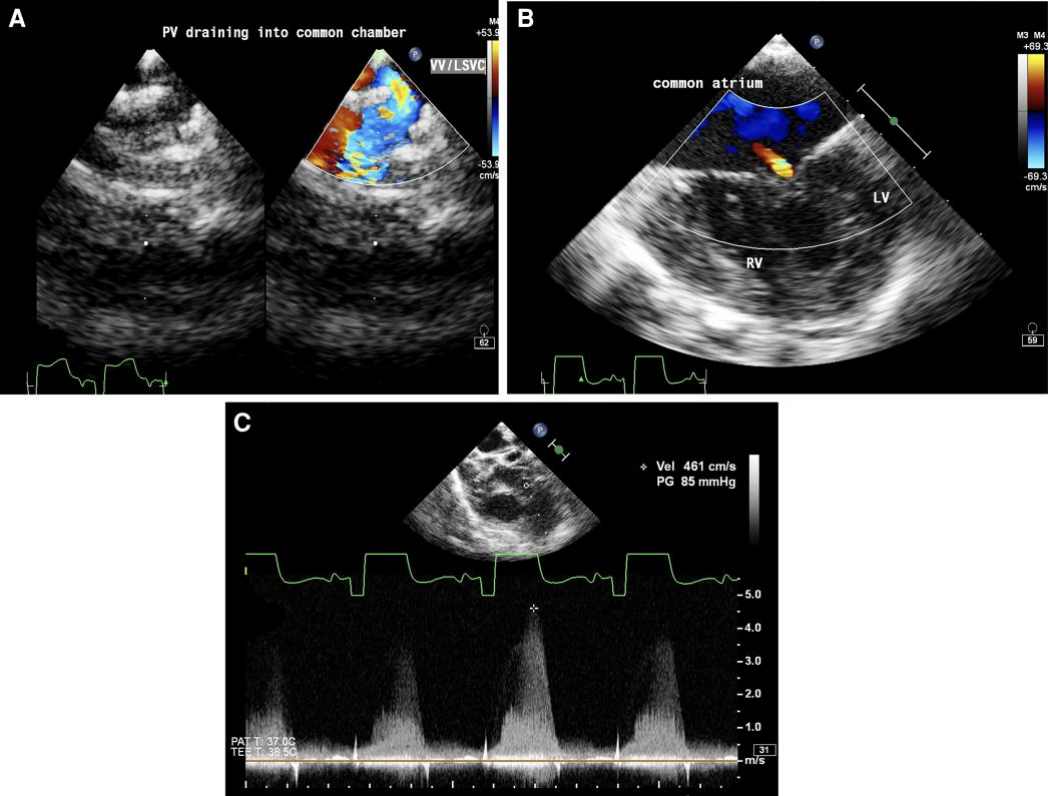
Transoesophageal echocardiogram (*A*) upper oesophageal view with colour Doppler showing pulmonary veins draining into vertical vein (VV)/left superior vena cava (LSVC). (*B*) Mid-oesophageal four-chamber view showing a common atrium, hypoplastic left ventricle (LV), well-formed right ventricle (RV), and mild common atrioventricular valve regurgitation. (*C*) Aortic short axis view with continuous wave Doppler across pulmonary valve showing pulmonary stenosis with a gradient of 85 mmHg.

The patient was planned for bilateral bidirectional Glenn shunt with TAPVC and AVVR repair under cardiopulmonary bypass (CPB). Intraoperative transoesophageal echocardiogram showed the presence of LSVC on a saline jet with a calibre similar to the right-sided vena cava with pulmonary veins draining into the LSVC (*Figure 3A–C*).

**Figure 4:**
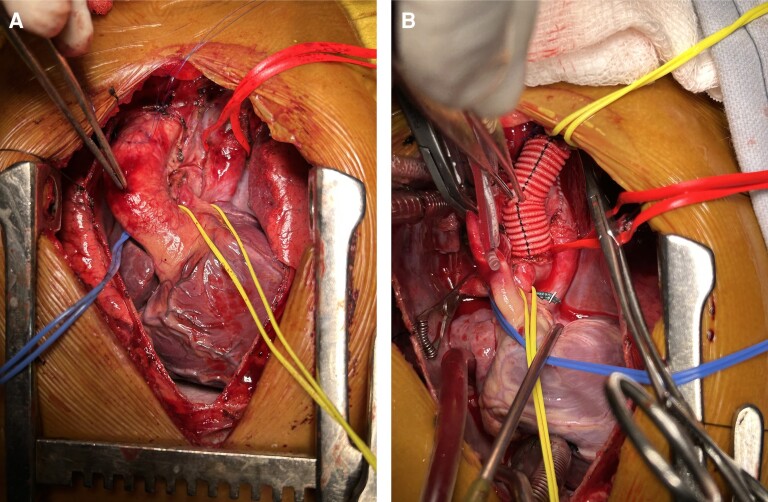
(*A*) Intraoperative photograph showing LSVC (red sling) with pulmonary veins draining into it, small MPA (yellow sling). (*B*) LSVC has been transected just below the junction of LSCV and LIJV and left-sided BD Glenn was done using an 8 mm Dacron tube graft. Pulmonary veins draining via LSVC/vertical vein into the common atrium (red sling).

A median sternotomy was done. The innominate vein was present but very small in calibre. The aorta was right, and anterior and the main pulmonary artery (MPA) was left and posterior. Branch pulmonary arteries were good-sized and confluent. Left internal jugular vein (LIJV) and left subclavian vein (LSCV) were found draining into LSVC that was then draining into the left atrium. Pulmonary veins of both sides were also draining into LSVC posteriorly via a common chamber, just below the confluence of LIJV and LSCV. Using aorto-bicaval cannulation, patient was taken on CPB and the heart was arrested using antegrade aortic root Delnido cardioplegia after cross-clamping the aorta. On opening the right atrium large ostium-primum atrial septal defect (ASD) was seen along with a single atrioventricular valve with mild AVVR. AV valve repair was done. Left superior vena cava was clamped and divided just distal to the junction of LSCV and LIJV and an 8 mm Dacron tube graft was anastomosed to divert blood from this junction to the left pulmonary artery. The pulmonary veins were left draining into the remaining part of LSVC that was draining into the left side of the common atrium (*Figure 4A* and *B*). The opening of LSVC and ASD was enlarged to provide for an unobstructed pulmonary venous flow. Right-sided vena cava was anastomosed to the right pulmonary artery in an end-to-side fashion. The right atrium was closed, and the patient was weaned off bypass.

Currently, the child is a year and a half post-surgery and is doing well, school going on par with the peer group maintaining a room air saturation of 87%.

## Discussion

Total anomalous pulmonary venous return is a congenital anomaly leading to cyanosis and left-to-right shunts (pre-cardiac), later compounded by pulmonary arterial hypertension (PAH). Consequently, it is considered a surgical emergency at the initial point of healthcare contact. Due to its propensity to be associated with other significant cardiac anomalies, detailed imaging becomes crucial for a comprehensive assessment, guiding tailored treatment for the specific lesion. In our presented case, the child exhibited numerous anomalies alongside a persistent LSVC.

Persistent left superior vena cava is a rare congenital venous anomaly draining into the right atrium via an enlarged coronary sinus, alternatively into the left atrium or a pulmonary vein. Lesions like large unrestricted ASD and VSD facilitate blood mixing within the chambers. In this case, the child had associated cardiac lesions, including DORV, VSD, complete AVCD with common atria, and PS, yet thrived well. Atrial septal defect and VSD allowed effective mixing, while PS prevented excessive blood flow to the pulmonary bed, thereby avoiding PAH.

Deoxygenated blood from systemic return mixed in the common atrium, facilitated by VSD. Blood from the left brachio-cephalic region drained through LSVC along with oxygenated blood from all pulmonary veins. This unique anatomical situation is illustrated in the accompanying diagram. Pulmonary stenosis directed a significant portion of the blood to the systemic circulation. The challenges in this case involved re-routing pulmonary and systemic venous drainage to their respective sides of the heart, considering the persistent LSVC and other anomalies.

In view of a challenging diagnostic scenario, the application of echocardiography led to the identification of supra cardiac TAPVC (with a vertical vein), which was contradicted by CT angiography revealing the existence of LSVC. Notably, the intraoperative findings revealed that the vertical vein and LSVC were the same structures, elucidating the correlation between the two diagnostic modalities. Due to this complex congenital anatomy, our surgical approach included ligating LSVC proximal to the pulmonary veins, left bidirectional Glenn with an 8 mm Dacron tube graft was performed (as shown in *Figure 4B*) as there was inadequate LSVC stump left after division, along with right bidirectional Glenn and AV valve repair. This anatomical re-routing yielded improved oxygenated blood to the common atrium, thereby enhancing saturation to systemic organs.

**Figure 5:**
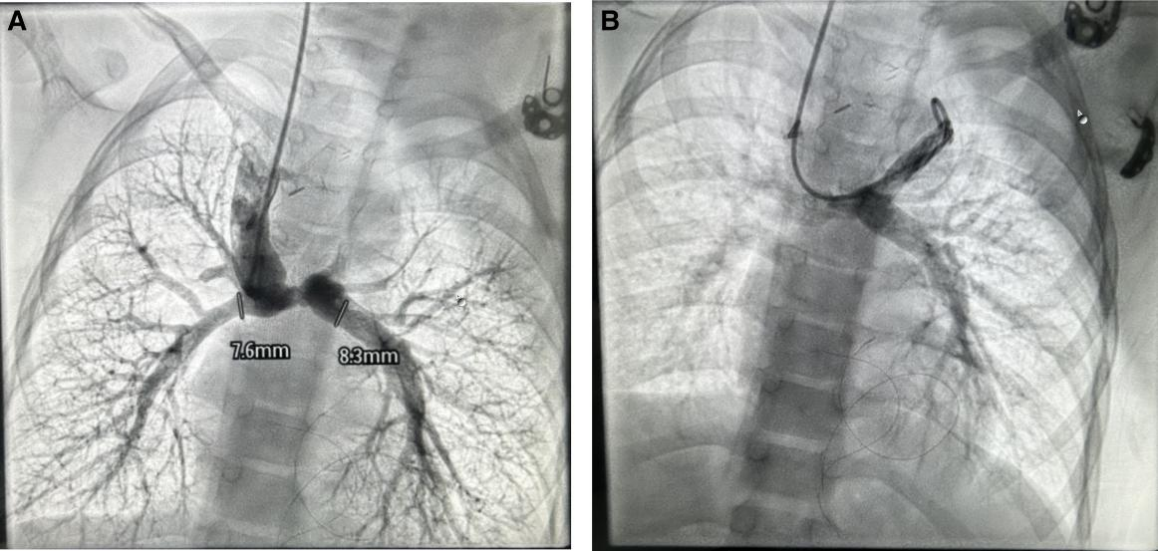
Catheterization study at 1-year follow-up (*A*) good flow in right BD Glenn with good arborization of the lung fields, MPA and LPA diameter mentioned (*B*) flow in left BG Glenn.

An e-PTFE graft was considered but as the patient was from low socio-economic background, it was deferred. One potential concern regarding patient growth pertains to the 8 mm tube graft, which may experience a relative decrease in size. It is noteworthy that this issue can be effectively rectified during the completion surgical procedure. At 6-month follow-up on echocardiogram good flows in bilateral BD Glenn shunts, good systemic ventricle function, good-sized branch PA’s, and trivial AVVR. At one-year follow-up, catheterization study was conducted to strategize for completion of TCPC that revealed good flow in right and left BD Glenn with good arborization with PA pressures of 14 mmHg (*Figure 5A* and *B*).

## Lead author biography


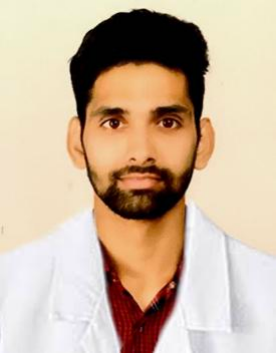


Dr R. Uday Tej, a driven and accomplished 30-year-old medical student, is currently pursuing senior residency in Cardiothoracic and Vascular Surgery (CTVS) at the esteemed institution, PGIMER. With a fervent dedication to the world of medicine and a particular interest in cardiothoracic surgery after. Finishing MBBS and MS from Kakatiya Medical College. Their research work showcases a deep commitment to advancing the field of CTVS and providing innovative solutions to medical challenges. Dr Uday’s academic journey reflects a promising career in health care, marked by a passion for learning and a desire to make a lasting impact in the medical community.

## Data Availability

The data underlying this article will be shared on reasonable request to the corresponding author.
